# Galectin-3 (MAC-2) Controls Microglia Phenotype Whether Amoeboid and Phagocytic or Branched and Non-phagocytic by Regulating the Cytoskeleton

**DOI:** 10.3389/fncel.2019.00090

**Published:** 2019-03-14

**Authors:** Fanny Reichert, Shlomo Rotshenker

**Affiliations:** Department of Medical Neurobiology, Institute for Medical Research Israel–Canada (IMRIC), Faculty of Medicine, Hebrew University, Jerusalem, Israel

**Keywords:** microglia, Galectin-3, phagocytosis, myelin, cytoskeleton, actin, cofilin, K-Ras

## Abstract

Myelin surrounding central nervous system (CNS) axons breaks down in multiple sclerosis (MS) and following traumatic axonal injury. Myelin-debris so produced is harmful to repair since it impedes remyelination in MS and the regeneration of traumatized axons. These devastating outcomes are largely due to inefficient removal by phagocytosis of myelin-debris by microglia. Therefore, revealing mechanisms that control phagocytosis is vital. We previously showed that in phagocytosis, filopodia and lamellipodia extend/engulf and then retract/internalize myelin-debris. Moreover, cofilin activates phagocytosis by advancing the remodeling of actin filaments (i.e., existing filaments disassemble and new filaments assemble in a new configuration), causing filopodia/lamellipodia to protrude, and furthermore, Galectin-3 (formally named MAC-2) activates phagocytosis by enhancing K-Ras.GTP/PI3K signaling that leads to actin/myosin-based contraction, causing filopodia/lamellipodia to retract. To understand further how Galectin-3 controls phagocytosis we knocked-down (KD) Galectin-3 expression in cultured primary microglia using Galectin-3 small-hairpin RNA (Gal-3-shRNA). KD Galectin-3 protein levels reduced phagocytosis extensively. Further, inhibiting nucleolin (NCL) and nucleophosmin (NPM), which advance K-Ras signaling as does Galectin-3, also reduced phagocytosis. Strikingly and unexpectedly, knocking down Galectin-3 resulted in a dramatic transformation of microglia morphology from “amoeboid-like” to “branched-like,” rearrangement of actin filaments and inactivation of cofilin. Thus, Galectin-3 may control microglia morphology and phagocytosis by regulating the activation state of cofilin, which, in turn, affects how actin filaments organize and how stable they are. Furthermore, our current and previous findings together suggest that Galectin-3 activates phagocytosis by targeting the cytoskeleton twice: first, by advancing cofilin activation, causing filopodia/lamellipodia to extend/engulf myelin-debris. Second, by advancing actin/myosin-based contraction through K-Ras.GTP/PI3K signaling, causing filopodia/lamellipodia to retract/internalize myelin-debris.

## Introduction

Microglia are a self-maintained population of innate immune cells unique to the central nervous system (CNS) parenchyma (Rio-Hortega, 1932; Sierra et al., 2016; Tay et al., 2016; Norris and Kipnis, 2018). Microglia differ by origin and molecular signature from CNS associated macrophages (i.e., perivascular, choroid plexus and meningeal) and from circulating monocytes (Ajami et al., 2007; Butovsky et al., 2014; Goldmann et al., 2016). Moreover, the intact blood brain barrier (BBB) separates microglia from CNS associated macrophages and circulating monocytes; yet, monocytes may enter the CNS parenchyma through dysfunctional BBB in trauma and disease (Mildner et al., 2007).

Microglia populate the developing CNS parenchyma during embryogenesis and further proliferate during the neonatal period. At that period, microglia display “amoeboid” morphology and phagocytic activity that enables them to remove apoptotic cells and eliminate/strip/prune synapses, shaping the CNS neuronal circuitry (Tremblay et al., 2010; Schafer et al., 2012; Kettenmann et al., 2013). Towards adulthood, microglia morphology transforms to “branched” and their phagocytic activity subsides. Upon injury and disease, microglia revert to amoeboid morphology and phagocytic activity resumes, enabling them to remove tissue debris (Rio-Hortega, 1932; Sierra et al., 2016; Norris and Kipnis, 2018). Thus, the notion that phagocytic activity correlates mostly with amoeboid morphology and non-phagocytic activity with branched morphology is long standing. Nonetheless, the molecular mechanisms that control the transformation from one phenotype to the other have remained largely unknown. It has been suggested that transcription factor Runx1 controls the *in vivo* postnatal conversion of forebrain microglia morphology from amoeboid to branched; yet, the involvement of Runx1 in phagocytosis was not tested (Zusso et al., 2012). It has further been shown that microglia were amoeboid and phagocytic when cultured in the presence of serum/FCS but branched and non-phagocytic when cultured in the absence of FCS; yet, the molecular mechanisms that induced each phenotype were not studied (Bohlen et al., 2017).

Our present study focuses on the phagocytosis of myelin-debris (often referred to as degenerated myelin). Myelin produced by oligodendrocytes surrounds CNS axons, enabling neuronal function through fast conduction of electrical activity. Myelin breaks down in demyelinating diseases such as multiple sclerosis (MS) and in Wallerian degeneration that traumatic axonal injury induces distal to lesion sites (e.g., spinal cord injury). Myelin-debris so produced is harmful to repair since it blocks remyelination in MS (Kotter et al., 2006; Lassmann et al., 2007) and impedes the regeneration/growth of traumatized axons (Yiu and He, 2006; Vargas and Barres, 2007). These devastating outcomes are largely due to inefficient removal by phagocytosis of myelin-debris, highlighting the significance of understanding mechanisms that control phagocytosis.

We previously showed that filopodia and lamellipodia extend/engulf and then retract/internalize myelin-debris in phagocytosis (Hadas et al., 2012). Mechanical forces generated by the cytoskeleton drive these structural changes. Protrusion of filopodia/lamellipodia requires that filaments of actin (F-actin) undergo remodeling, i.e., existing F-actin disassemble and new F-actin assemble in a new configuration, causing plasma membranes to protrude (Oser and Condeelis, 2009; Bernstein and Bamburg, 2010). We previously showed that cofilin, a member of the actin depolymerizing factor (ADF) family that advances filopodia/lamellipodia production by disassembling F-actin, activates phagocytosis (Hadas et al., 2012; Gitik et al., 2014), and further, that actin/myosin-based contraction drives filopodia/lamellipodia to retract/internalize myelin-debris (Gitik et al., 2010).

We further previously suggested two mechanisms that impede the phagocytosis of myelin-debris. In the first, myelin-debris itself attenuates its own phagocytosis. In this regard, CD47 on myelin binds SIRPα (CD172a) on microglia and macrophages, and in turn, SIRPα generates “don’t eat me” signaling in which cofilin is inactivated, the remodeling of F-actin is obstructed, and phagocytosis is reduced (Gitik et al., 2011, 2014). This could be the case in MS since the removal by phagocytosis of myelin-debris is inefficient in MS (Kotter et al., 2006; Lassmann et al., 2007). The second mechanism could play a role in CNS Wallerian degeneration (i.e., distal to but not including the lesion site), where microglia fail to phagocytose myelin-debris altogether. We suggested that this failure results mostly from microglia failing to upregulate the expression of the β-galactoside-binding lectin Galectin-3 (formally named MAC-2; Rotshenker et al., 2008; Rotshenker, 2009).

Many normal and malignant cells produce and secrete Galectin-3, a member of a large family of galectins. Galectin-3 takes part in numerous functions in health and disease; e.g., pre-mRNA splicing in the nucleus, signaling pathways in cytoplasm, and activation of surface receptors extracellularly (Ruvolo, 2016; Thiemann and Baum, 2016; Mèndez-Huergo et al., 2017). Amongst functions that relate to our current project, Galectin-3 advances K-Ras signaling in the cytoplasm. K-Ras is a member of the Ras family of small GTPases K-, H- and N-Ras that are active when GTP bound and inactive when GDP bound. Galectin-3 binds and stabilizes active K-Ras.GTP at the inner surface of cell membranes, prolonging K-Ras dependent signaling (Tian et al., 2010). Of further interest to us are nucleolin (NCL) and nucleophosmin (NPM) since, amongst their various functions, the two advance K-Ras dependent signaling by chaperoning and stabilizing K-Ras.GDP at the inner surface of plasma membranes where it needs to be activated (Inder et al., 2010). Further, NCL is present at low levels in plasma membranes of some normal cells (e.g., microglia) and at much higher levels in some malignant cells (Hirano et al., 2005; Bates et al., 2009; Ozawa et al., 2013). As a cell surface receptor, NCL binds and is instrumental in endocytosing particulate material.

We previously showed that Galectin-3 expression correlates with myelin-debris phagocytosis in microglia. In adult mice, non-phagocytosing microglia did not express Galectin-3 in intact CNS nor in CNS Wallerian degeneration, i.e., distal to but not including the lesion site. In contrast, and along with activating phagocytosis, microglia upregulated Galectin-3 expression *in vivo* at lesion sites and in experimental allergic encephalomyelitis (EAE), and in cultured primary microglia (Reichert and Rotshenker, 1996, 1999). We further showed that Galectin-3 activated phagocytosis by binding and stabilizing K-Ras.GTP, prolonging the activity of K-Ras.GTP/PI3K signaling (Rotshenker et al., 2008; Rotshenker, 2009), leading to PI3K/PLC/PKC signaling (Makranz et al., 2004; Cohen et al., 2006), and then to actin/myosin-based contraction (Gitik et al., 2010).

Our aims in this project have been to further understand and validate the role of Galectin-3 as a key regulator of myelin-debris phagocytosis. First, we infected cultured primary microglia with Galectin-3 small-hairpin RNA (Gal-3-shRNA) to reduce/knockdown (KD) Galectin-3 protein levels, generating Gal-3-KD microglia. Second, we aimed to determine whether NCL and NPM activate phagocytosis as predicted from their ability to advance K-Ras signaling as Galectin-3 does. Third, we aimed to reveal which cytoskeletal molecules regulated by Galectin-3 activate phagocytosis. Our current findings suggest that Galectin-3 controls both microglia morphology and phagocytosis by targeting the cytoskeleton.

## Materials and Methods

### Animals

Balb/C wild-type mice (Harlan Sprague-Dawley, Inc., Israel) and transgenic Galectin-3 knockout (Gal-3^−/−^) mice (provided by Prof. Yoel Kloog; Levy et al., 2011) were used in accordance with the National Research Council’s guide for the care and use of laboratory animals and with the approval of the institutional ethics committee Hebrew University Faculty of Medicine.

### Media Products

DMEM, DMEM/F12, FCS, HI-FCS, Gentamicin sulfate and L-Glutamine obtained from Biological Industries (Beit-Haemek, Israel).

### Isolation of Primary Microglia

Microglia were isolated from brains of neonate mice as previously described (Reichert and Rotshenker, 2003). In brief, brains were stripped of their meninges, enzymatically dissociated, cells plated on poly-L-lysine coated flasks for 1 week, replated for 1- to 2-h on bacteriological plates and non-adherent cells washed away. The vast majority of adherent cells are microglia judged by morphology and expression of P2Y12 (Butovsky et al., 2014), and Galectin-3, complement receptor-3 (CR-3) and F4/80 (Reichert and Rotshenker, 1996, 1999). Microglia were maintained in DMEM/10% HI-FCS and 10% medium conditioned by the L-cell line that produces CSF-1 (American Type Culture Collection, Rockville, VA, USA).

### Generation of Microglia With Stable Reduced Galectin-3 Protein Expression

Knocking down Galectin-3 protein expression was achieved through lentiviral infection of wild-type Balb/C microglia with shRNA directed against mouse Galectin-3 mRNA (Gal-3-shRNA) using pLKO.1 puro plasmids (Sigma-Aldrich, St. Louis, MO, USA). We tested three different shRNA sequences and finally used sequence 5′-GCAGTACAACCATCGGATGAA-3′. The plasmid was transfected into a 293T-based packaging cell line and the resulting culture supernatant used for lentiviral infection. Infected microglia were selected based on their resistance to puromycin brought by the pLKO.1 plasmid and levels of Galectin-3 protein were monitored by immunoblot. We refer to these microglia as Gal-3-KD microglia. As a control, microglia were infected in a similar way with the shRNA sequence 5′-CTTACGCTGAGTACTTCGA-3′ against the non-target firefly Luciferase gene. We refer to these microglia as control or control Luciferase (Con-Luc) microglia.

### Isolation of Myelin

Myelin isolation from mouse brains was performed as previously described (Slobodov et al., 2001) and visualized (Gitik et al., 2010). Isolated myelin is “myelin-debris” since isolation involves breakdown of intact myelin.

### Phagocytosis of Myelin-Debris

Microglia were plated in 96-well tissue culture plates at a density that minimizes cell-cell contact (0.25–1.5 × 10^4^/well) in the presence of DMEM/F12 supplemented by 10% FCS. Non-adherent microglia were washed out after 2-h and adherent microglia left to rest overnight. Next, phagocytes were washed and myelin-debris added in the presence of 10% FCS for the indicated periods, unphagocytosed myelin-debris washed out, and levels of phagocytosis determined by ELISA. For testing the role of NCL and NPM in phagocytosis, microglia were pre-incubated overnight in 10 μM NCL inhibitor GRO (AS1411) aptamer and its control CRO (inactive oligomer; Integrated DNA Technologies, Coralville, IA, USA) or 2-h in 4 μM of NPM inhibitor NSC348884 (Cayman Chemicals, Ann Arbor, MI, USA). Then, 1-h of phagocytosis was assayed.

### Quantifying Myelin-Debris Phagocytosis by ELISA

We quantified phagocytosis as previously detailed (Slobodov et al., 2001). The assay is based on the detection of myelin basic protein (MBP) in microglia lysates. Since MBP is unique to myelin and not produced by microglia, MBP levels in microglia cytoplasm are proportional to levels of phagocytosed myelin-debris. In brief, after washing unphagocytosed myelin-debris, microglia were lysed (0.05 M carbonate buffer, pH 10), lysates transferred to high protein absorbance plates (Nalge Nunc International, Rochester, NY, USA) and levels of MBP determined by ELISA using rat anti-MBP mAb and matching control IgG (Bio-Rad Laboratories Inc., Hercules, CA, USA).

When phagocytosis by Gal-3-KD microglia was compared to phagocytosis by control (Con-Luc) microglia, phagocytosis by each population was first normalized to the respective number of microglia counted in 1-mm^2^ area at the center of wells. Normalizing phagocytosis to cell number is required since Gal-3-KD and control microglia may differ in their adherence properties, thus resulting in different number of adherent microglia even when the same number of cells was initially seeded. To this end, microglia in replicate plates were fixed, stained and counted. Phagocytosis by Gal-3-KD microglia was calculated as percentage of phagocytosis by control microglia normalized to 100%. Phagocytosis in the presence of NCL and NPM inhibitors was calculated as percentage of phagocytosis in the presence of their respective controls, each control normalized to 100%.

### Immunoblot Analysis

Microglia were plated in 10-cm tissue culture plates at a density that minimizes cell-cell contact (3 × 10^6^ cells per plate) in the presence of DMEM supplemented by 10% FCS, and left to rest overnight. Phagocytes were washed in fresh DMEM supplemented by 10% FCS, myelin-debris added in the presence of serum for the indicated periods and unphagocytosed myelin-debris washed out. For lysis, microglia were washed in PBS and lysed in ice-cold lysis buffer (Tris HCL 1 M pH 7.5, MgCl_2_ 1 M, NaCl 4 M, 0.5% NP-40, 0.1% DTT, 0.1% NaVa), supplemented with protease and phosphatase inhibitors cocktails (Sigma-Aldrich, St. Louis, MO, USA), cellular debris was removed by centrifugation, and total protein content determined using Bradford reagent (Sigma-Aldrich, St. Louis, MO, USA). Equal protein content from whole cell lysates was separated on SDS-PAGE. Proteins were blotted to nitrocellulose membranes, blocked with 10% non-fat milk or 5% BSA in Tris-buffered saline (TBS) for 1-h at RT, incubated over night at 4^0^C in the presence of rat anti-mouse Galectin-3/MAC-2 M3/38 mAb (American Type Collection, Rockville, MD, USA), rabbit anti-cofilin, rabbit anti-pS^3^-cofilin-1, and rabbit anti-β-tubulin (Santa Cruz Biotechnology, Santa Cruz, CA, USA). Blots were washed with TBST and incubated with respective secondary Abs donkey anti-rat and goat anti-rabbit conjugated to HRP (Jackson ImmunoResearch, West Grove, PA, USA) for 40-min at RT. Proteins were visualized with EZ-ECL kit for HRP detection (Beit Haemek, Israel). The intensities of immunoblot bands were determined by ImageJ software.

### Confocal Fluorescence Microscopy

Microscopy was carried out in Olympus FluoView FV1000 confocal microscope. Alexa Fluor 488 labeled phalloidin (Invitrogen, Carlsbad, CA, USA) was used to visualize F-actin. CR3 was visualized using two rat anti-mouse CR3 mAbs produced by hybridoma cell lines: mAb M1/70 (Developmental Studies Hybridoma Bank, Iowa City, IA, USA) and mAb 5C6 (American Type Culture Collection, Rockville, VA, USA) followed by Cy3-conjugated rabbit anti-rat IgG (Jackson ImmunoResearch, West Grove, PA, USA). Optical slices, 1 μm thick, were scanned sequentially and then used to reconstruct whole images.

### Statistical Analysis and Data Presentation

Parametric statistics were used after verifying that all data values follow a Gaussian distribution. The D’Agostino-Pearson normality test, unpaired *t*-test and one- and two way ANOVA were carried out using GraphPad Prism software as detailed in figure legends.

## Results

### Galectin-3 Activates the Phagocytosis of Myelin-Debris

**Figure 1:**
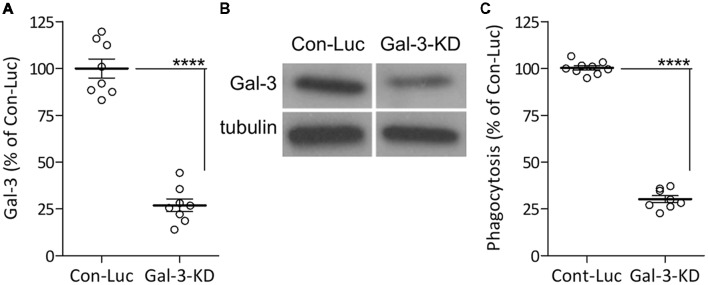
The phagocytosis of myelin-debris is reduced in microglia expressing reduced levels of Galectin-3 protein. **(A,B)** Galectin-3 (Gal-3) protein levels were reduced/knocked-down (KD) in microglia infected with Gal-3-small-hairpin RNA (shRNA; Gal-3-KD microglia) compared with control microglia infected with non-target Luciferase-shRNA (Con-Luc microglia). **(A)** Quantification of Gal-3 protein levels based on **(B)** immunoblot analysis. **(A)** Gal-3 levels in Gal-3-KD microglia were calculated as percentage of Gal-3 levels in control microglia (Con-Luc) normalized to 100%. Values from individual blots and averages ± SE are given. Significance of difference, ****p < 0.0001, by unpaired *t*-test. **(C)** Phagocytosis of myelin-debris is reduced in Gal-3-KD microglia. Phagocytosis by Gal-3-KD microglia was calculated as percentage of phagocytosis by control microglia (Con-Luc) normalized to 100%. Values of individual experiments and averages ± SE are given. Significance of difference, ****p < 0.0001, by unpaired *t*-test.

We previously showed that Galectin-3 activates the phagocytosis of myelin-debris in cultured primary microglia by binding and stabilizing K-Ras.GTP and so enhancing K-Ras.GTP/PI3K signaling (Rotshenker et al., 2008; Rotshenker, 2009). We reached this understanding based on the findings that levels of K-Ras. GTP alone and levels of the K-Ras.GTP/Galectin-3 complex increased during phagocytosis, and further, that disrupting the K-Ras.GTP/Galectin-3 complex pharmacologically reduced K-Ras.GTP levels, PI3K activity and phagocytosis. To validate the key role Galectin-3 plays in activating phagocytosis, we presently KD Galectin-3 protein levels in cultured primary microglia through lentiviral infection with Gal-3-shRNA, generating Gal-3-KD microglia. We used microglia infected with non-target Luciferase-shRNA as control. Gal-3-KD microglia displayed about 70% reductions in both Galectin-3 protein levels and myelin-debris phagocytosis compared with control microglia (Figure 1). This finding conforms to our previous observations that disrupting the K-Ras.GTP/Galectin-3 complex pharmacologically reduced phagocytosis by 70% (Rotshenker et al., 2008). Thus, our present and previous findings validate the key role that Galectin-3 plays in activating phagocytosis.

### Nucleolin (NCL) and Nucleophosmin (NPM) Activate the Phagocytosis of Myelin-Debris

**Figure 2:**
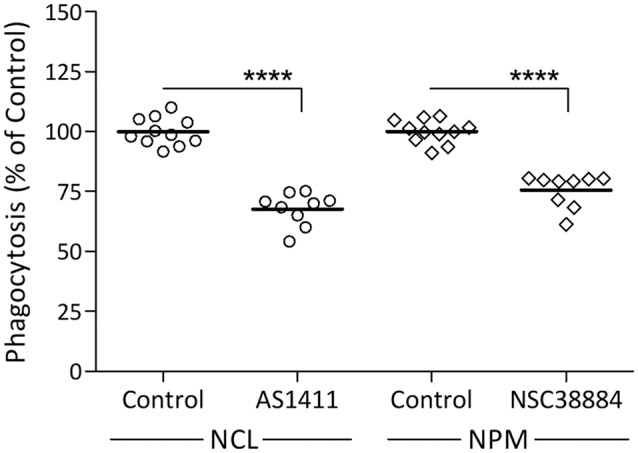
Nucleolin (NCL) and nucleophosmin (NPM) inhibitors reduce the phagocytosis of myelin-debris. Wild-type microglia were pre-incubated in NCL inhibitor AS1411, NPM inhibitor NSC38884 and their respective controls, myelin-debris added for 1-h and phagocytosis assayed. Phagocytosis in the presence of each inhibitor was calculated as percentage of phagocytosis in its respective control normalized to 100%. Values of individual experiments and averages ± SE are given. Significance of difference, ****p < 0.0001, by unpaired *t*-test.

Our previous findings suggest that Galectin-3 activates the phagocytosis of myelin-debris by advancing K-Ras signaling (Rotshenker et al., 2008; Rotshenker, 2009). Since NCL and NPM also advance K-Ras signaling (Inder et al., 2010), we hypothesized that NCL and NPM could activate phagocytosis as does Galectin-3. We tested this prediction using specific NCL and NPM inhibitors (Figure 2). NCL inhibitor AS14111 reduced phagocytosis by about 30% and NPM inhibitor NSC38884 reduced phagocytosis by about 25%. This contrasts with about 70% reduced phagocytosis caused by knocking down Galectin-3 protein levels in Gal-3-KD microglia (Figure 1) and about 70% reduced phagocytosis caused by disrupting the K-Ras.GTP/Galectin-3 complex pharmacologically (Rotshenker et al., 2008; Rotshenker, 2009). This discrepancy could result, in part, from the different mechanism by which the three advance K-Ras signaling. Galectin-3 does so by stabilizing K-Ras.GTP (i.e., already activated K-Ras) and NCL and NPM by stabilizing K-Ras.GDP that yet needs to undergo activation at the inner surface of cell membranes (Inder et al., 2010; Tian et al., 2010).

### Galectin-3 Controls Microglia Morphology and the Organization of Actin Filaments

**Figure 3:**
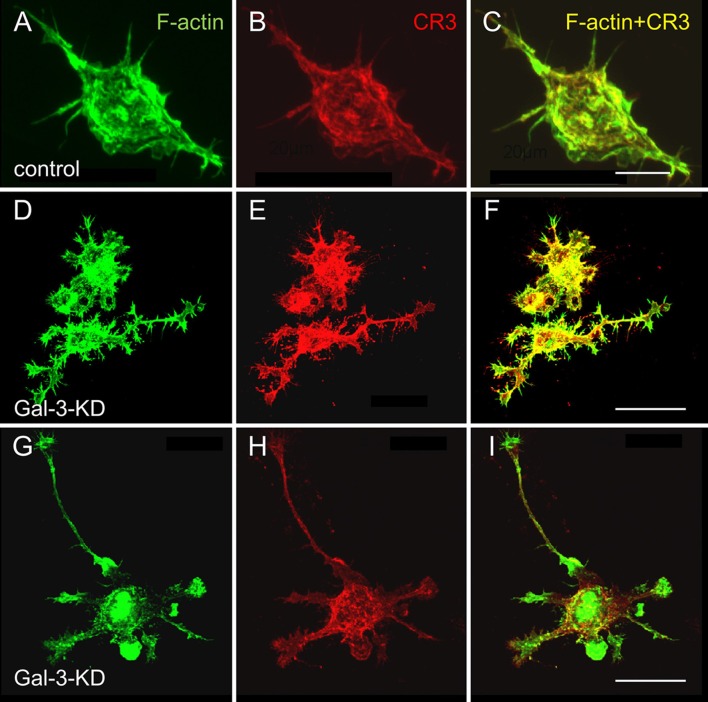
Control microglia display amoeboid-like morphology and organization of filaments of actin (F-actin) in filamentous structures whereas Gal-3-KD microglia display branched-like morphology and organization of F-actin in dense punctate and massive deposits. Immunofluorescence confocal microscopy images of **(A–C)** a control microglia and **(D** through** I)** two fields of Gal-3-KD microglia. **(A,D,G)** F-actin is visualized by Alexa Fluor 488 labeled phalloidin (green). **(B,E,H)** Complement receptor-3 (CR3), a principal phagocytic receptor that mediates the phagocytosis of myelin-debris (Rotshenker, 2003), is visualized by anti-CR3 mAbs M1/70 and 5C6 (red). **(C,F,I)** F-actin/CR3 overlap (yellow). As seen, the images of Gal-3-KD microglia in (**D** through** I**) are reduced by a factor of five compared with the images of the control microglia in **(A–C)**. Bars: 10 μm in **(C)** for control microglia and 40 μm in **(F,I)** for Gal-3-KD microglia.

Strikingly and unexpectedly, the morphology of cultured Gal-3-KD microglia differed dramatically from that of cultured control microglia (Figure 3). Control microglia were mostly oval/ellipsoid and fine filopodia projected from them. In contrast, Gal-3-KD microglia extended thick branches that varied from short to very long. Some of these primary branches gave rise to secondary thick branches and filopodia projected from both primary and secondary branches. The morphology of control and Gal-3-KD microglia was very similar to that of cultured microglia obtained from wild-type and Galectin-3 knockout (Gal-3^−/−^) mice (Figure 5). We refer to the morphology of Gal-3-KD microglia as being “branched-like” and that of control microglia as being “amoeboid-like” since the two reminded us of the “branched” and “amoeboid” morphologies described by Rio-Hortega (1932) in the intact and injured CNS parenchyma, respectively (Rio-Hortega, 1932; Sierra et al., 2016).

**Figure 4:**
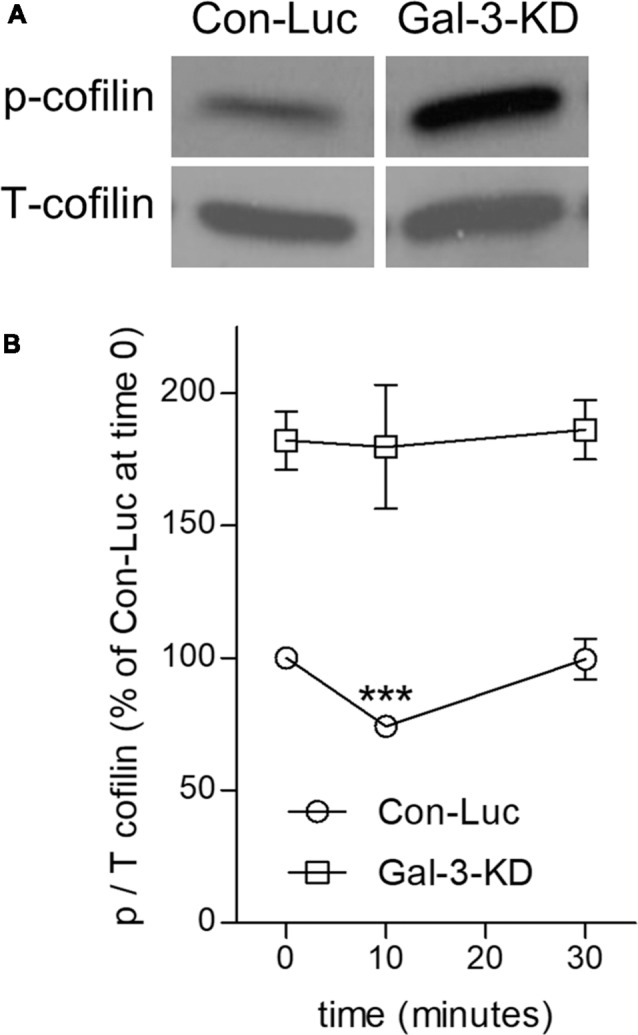
Knocking down Galectin-3 advances the inactive state of cofilin (cofilin → p-cofilin) in non-phagocytosing and phagocytosing Galectin-3-KD microglia. **(A)** Levels of inactive p-cofilin are higher in non-phagocytosing Gal-3-KD than in non-phagocytosing control (Con-Luc) microglia. A representative immunoblot (one of six) of phosphorylated and total cofilin-1 (p- and T-cofilin). The western blot images were cropped for illustration purposes in Supplementary Figure S1. **(B)** Cofilin is transiently activated (i.e., transient reduction in p-cofilin) in control (Con-Luc) microglia but not in Gal-3-KD microglia. Quantitation of the ratio p/T cofilin based on immunoblots of microglia lysates taken before (time 0) and after 10 and 30 min of phagocytosis. The ratio p/T cofilin in non-phagocytosing control (Con-Luc) microglia (time 0 as in **A**) was defined 100%. Then, p/T in all other non-phagocytosing and phagocytosing microglia was calculated as percentage of p/T in control (Con-Luc) microglia at time 0. Average values ± SEM of six experiments, each performed in duplicates, are given. Significance of differences between initial values at 0 min and those at 10 and 30 min was calculated for control (Con-Luc) and Gal-3-KD microglia separately by one-way ANOVA and the Dunnett post-test. Activation of cofilin is significant, ***p < 0.001, in control (Con-Luc) microglia at 10 min. The difference between control (Con-Luc) and Gal-3-KD microglia is significant at all-time points (0, 10 and 30 min), *p* < 0.001 for each time point, by two way ANOVA and the Bonferroni posttest (not marked on the graph).

**Figure 5:**
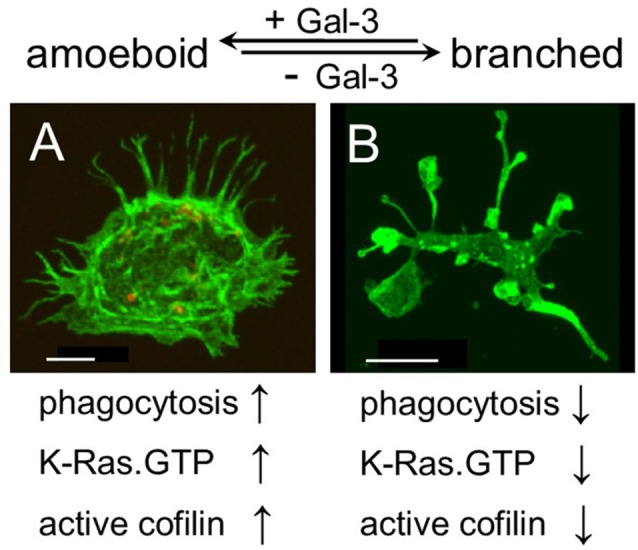
Galectin-3 controls microglia phenotype whether amoeboid and phagocytic or branched and non-phagocytic by regulating the cytoskeleton. Immunofluorescence confocal microscopy images of cultured **(A)** wild-type and **(B)** Gal-3^−/−^ microglia. F-actin is visualized by Alexa Fluor 488 labeled phalloidin (green). As seen, the image of the Gal-3^−/−^ microglia in **(B)** is reduced by a factor of 6.4 compared with the image of the wild-type microglia in **(A)**. Bars: 5 μm in **(A)** and 20 μm in **(B)**.

Moreover, the organization of actin filaments (F-actin) in control microglia differed strikingly from that in Gal-3-KD microglia (Figure 3). In control microglia, F-actin appeared in fine filamentous structures at the cell center and in closely packed filamentous structures at the cell cortex. In contrast, in Gal-3-KD microglia, F-actin appeared predominantly in highly dense deposits that varied from punctate to massive, and furthermore, fine filamentous structures were barely detected, if at all. The organization of F-actin in control and Gal-3-KD microglia was similar to that in cultured microglia from wild-type and Gal-3^−/−^ mice (Figure 5). Thus, knocking down and knocking out Galectin-3 resulted in a dramatic change in both the morphology of microglia and the organization of F-actin in microglia, raising the possibility that Galectin-3 controls microglia morphology by regulating the organization of F-actin.

### Galectin-3 Advances the Activation of Cofilin

Changing the organization of F-actin in cells requires that existing F-actin disassemble and new F-actin assemble in a different configuration from before (i.e., remodeling of F-actin). Active unphosphorylated cofilin (cofilin) initiates remodeling by causing F-actin to disassemble whereas inactive phosphorylated cofilin (p-cofilin) advances F-acting stabilization by obstructing the disassembly of F-actin (Oser and Condeelis, 2009; Bernstein and Bamburg, 2010). This understanding led us to hypothesize that cofilin could be instrumental in changing the organization of F-actin in control and wild-type microglia from that in Gal-3-KD and Gal-3^−/−^ microglia (Figures 3, 5). Further, we showed before that active cofilin activated and inactive p-cofilin inhibited phagocytosis in which filopodia/lamellipodia engulf myelin-debris (Hadas et al., 2012; Gitik et al., 2014). Taken all in consideration, could Galectin-3 control microglia morphology, F-actin organization, and phagocytosis by regulating the activation state of cofilin?

We addressed this issue by analyzing the activation state of cofilin before and during phagocytosis in control and Gal-3-KD microglia (Figure 4). Levels of inactive cofilin (i.e., p-cofilin), which advances the stabilization of F-actin, were higher in non-phagocytosing Gal-3-KD microglia than in non-phagocytosing control microglia. Then, during phagocytosis, in agreement with our previous findings (Hadas et al., 2012; Gitik et al., 2014), control microglia displayed transient activation of cofilin (i.e., transient reduction in p-cofilin levels). In contrast, levels of inactive p-cofilin remained high throughout phagocytosis in Gal-3-KD microglia. Taken altogether, it is very likely that knocking down Galectin-3 prompted structural changes in non-phagocytosing Gal-3-KD microglia and further reduced their phagocytic activity by advancing the inactive state of cofilin (i.e., increasing p-cofilin levels).

## Discussion

Our findings are the first to suggest a molecular mechanism that controls both the morphology and the phagocytic activity of microglia, namely, that Galectin-3, by regulating the cytoskeleton, controls microglia phenotype whether amoeboid and phagocytic or branched and non-phagocytic. We reached this understanding based on the findings that amoeboid microglia were rich in Galectin-3, they displayed productive phagocytosis and F-actin organized in them in filamentous structures readily accessible for remodeling by active cofilin. In contrast, branched microglia were deficient in Galectin-3, they displayed unproductive phagocytosis, F-actin organized in them in punctate and/or massive deposits stabilized by inactive cofilin and the appearance of F-actin in filamentous structures was scarce. Thus consistent with the realization that the cytoskeleton controls cell morphology, the presence or absence of Galectin-3 could determine microglia morphology, whether amoeboid or branched, by controlling the activation state of cofilin and so affecting how F-actin is organized and how stable any particular organization is. Consistent with the realization that cofilin driven remodeling of F-actin advances the protrusion of filopodia/lamellipodia (Oser and Condeelis, 2009; Bernstein and Bamburg, 2010) and our previous findings that cofilin activates phagocytosis in which filopodia/lamellipodia engulf myelin-debris (Hadas et al., 2012; Gitik et al., 2014), Galectin-3 could activate phagocytosis by advancing the activation of cofilin. Taken altogether, it is most probable that Galectin-3 controls microglia phenotype whether amoeboid/phagocytic or branched/non-phagocytic by regulating the activation state of cofilin, which, in turn, affects the organization and stability of F-actin (Figure 5).

Galectin-3 activating the phagocytosis of myelin-debris through cofilin, as we show here, adds to our previous findings that Galectin-3 activated phagocytosis by enhancing K-Ras.GTP/PI3K signaling (Rotshenker et al., 2008; Rotshenker, 2009), leading to PI3K/PLC/PKC signaling (Makranz et al., 2004; Cohen et al., 2006) and then to actin/myosin-based contraction, causing filopodia/lamellipodia to retract/internalize myelin-debris (Gitik et al., 2010). Our present findings that NCL and NPM activated phagocytosis, albeit less than Galectin-3, agree with and further support the concept that Galectin-3 activates phagocytosis by advancing K-Ras signaling since also NCL and NPM advance K-Ras signaling (Inder et al., 2010; Tian et al., 2010). Nonetheless, as we currently show, Galectin-3 dominated over NCL and NPM in this regard. Thus, our present and previous findings together suggest that Galectin-3 activates phagocytosis by targeting the cytoskeleton twice: first, by advancing the active state of cofilin, leading to F-actin remodeling, and second, by advancing K-Ras.GTP/PI3K signaling, leading to actin/myosin-based contraction (Figure 5).

It is most probable that our proposition that Galectin-3 controls microglia phenotype whether amoeboid/phagocytic or branched/non-phagocytic, which we base on findings in cultured microglia, applies to the *in vivo* scenario. The concept of Galectin-3 activated phagocytosis is supported by our previous findings that the expression of Galectin-3 (named MAC-2 in some of our previous studies) correlated with myelin-debris phagocytosis in microglia *in vivo*. In adult mice, non-phagocytosing microglia did not express Galectin-3 protein in intact CNS nor in CNS Wallerian degeneration, i.e., distal to lesion sites. In contrast, and along with activating phagocytosis, microglia upregulated Galectin-3 expression at sites of trauma and in EAE (Reichert and Rotshenker, 1996, 1999). With regard to Galectin-3 controlling microglia morphology, two reports are of interest to us. First, that transcription factors Runx1 and Runx2 regulate Galectin-3 expression in human pituitary tumors (Zhang et al., 2009). Second, that amoeboid microglia in neonate mice but not branched microglia in adult mice expressed Runx1, and further, that Runx1 is upregulated in spinal cord microglia activated by nerve injury in adult mice (Zusso et al., 2012). If in mice, as in human pituitary tumors, Runx1 controls Galectin-3 protein expression, then Runx1 controls microglia morphology *in vivo* through Galectin-3 as our present findings suggest. Lastly, Galectin-3 may also be involved in the pruning of synapses during the neonatal period and in the early loss of synapses in Alzheimer diseases (Schafer et al., 2012; Hong et al., 2016). We base this suggestion on the findings that in these two instances CR3 mediated the phagocytosis of synaptic elements in microglia and that CR3 is the principal phagocytic receptor that mediated the phagocytosis of myelin-debris in microglia in our studies (Reichert and Rotshenker, 2003; Rotshenker, 2003). Thus, Galecin-3 could activate CR3 mediated phagocytosis of synaptic elements as it activated CR3 mediated phagocytosis of myelin-debris. Taken altogether, Galectin-3 may control microglia morphology and phagocytic activity *in vivo* as *in vitro*.

## Author Contributions

SR designed and supervised experiments conducted by FR.

## Conflict of Interest Statement

The authors declare that the research was conducted in the absence of any commercial or financial relationships that could be construed as a potential conflict of interest.

## Supplementary Material

FIGURE S1 Levels of inactive p-cofilin are higher in non-phagocytosing control (Con-Luc)microglia than in non-phagocytosing Gal-3-KD microglia. **(A,B)** Two separateimmunoblots were carried out using replicate lysate samples from non-phagocytosing control(Con-Luc) and Gal-3-KD microglia. In **(A)**, p-cofilin was visualized and in **(B)**, T-cofilin wasvisualized. The marked images were cropped and used for illustration in manuscript Figure 4.
